# Compatibilization of Polylactide/Poly(ethylene 2,5-furanoate) (PLA/PEF) Blends for Sustainable and Bioderived Packaging

**DOI:** 10.3390/molecules27196371

**Published:** 2022-09-27

**Authors:** Giulia Fredi, Andrea Dorigato, Alessandro Dussin, Eleftheria Xanthopoulou, Dimitrios N. Bikiaris, Luigi Botta, Vincenzo Fiore, Alessandro Pegoretti

**Affiliations:** 1 Department of Industrial Engineering and INSTM Research Unit, University of Trento, Via Sommarive 9, 38123 Trento, Italy; 2 Chemistry Department, Laboratory of Polymer Chemistry and Technology, Aristotle University of Thessaloniki, 54124 Thessaloniki, Greece; 3 Department of Engineering, University of Palermo, Viale delle Scienze, 90128 Palermo, Italy

**Keywords:** polylactide, furanoates, poly(ethylene furanoate), blends, compatibilization, gas-permeability, UV-shielding

## Abstract

Despite the advantages of polylactide (PLA), its inadequate UV-shielding and gas-barrier properties undermine its wide application as a flexible packaging film for perishable items. These issues are addressed in this work by investigating the properties of melt-mixed, fully bioderived blends of polylactide (PLA) and poly(ethylene furanoate) (PEF), as a function of the PEF weight fraction (1–30 wt %) and the amount of the commercial compatibilizer/chain extender Joncryl ADR 4468 (J, 0.25–1 phr). J mitigates the immiscibility of the two polymer phases by decreasing and homogenizing the PEF domain size; for the blend containing 10 wt % of PEF, the PEF domain size drops from 0.67 ± 0.46 µm of the uncompatibilized blend to 0.26 ± 0.14 with 1 phr of J. Moreover, the increase in the complex viscosity of PLA and PLA/PEF blends with the J content evidences the effectiveness of J as a chain extender. This dual positive contribution of J is reflected in the mechanical properties of PLA/PEF blends. Whereas the uncompatibilized blend with 10 wt % of PEF shows lower mechanical performance than neat PLA, all the compatibilized blends show higher tensile strength and strain at break, while retaining their high elastic moduli. The effects of PEF on the UV- and oxygen-barrier properties of PLA are also remarkable. Adding only 1 wt % of PEF makes the blend an excellent barrier for UV rays, with the transmittance at 320 nm dropping from 52.8% of neat PLA to 0.4% of the sample with 1 wt % PEF, while keeping good transparency in the visible region. PEF is also responsible for a sensible decrease in the oxygen transmission rate, which decreases from 189 cc/m^2^·day for neat PLA to 144 cc/m^2^·day with only 1 wt % of PEF. This work emphasizes the synergistic effects of PEF and J in enhancing the thermal, mechanical, UV-shielding, and gas-barrier properties of PLA, which results in bioderived blends that are very promising for packaging applications.

## 1. Introduction

Bioplastics, i.e., plastics that are biodegradable and/or derived from renewable resources [1], may represent a promising alternative to conventional plastics for several applications. Although plastics have become essential for daily and high-end applications thanks to their exceptional versatility, good processability, outstanding mechanical and functional properties, and low cost [2], their petrochemical origin and non-biodegradability can represent a serious environmental threat. On the other hand, bioderived bioplastics allow a substantial reduction in the carbon footprint in the stage of resource extraction, while biodegradable bioplastics have alternative routes for waste disposal, thus limiting the quantity of plastic waste ending up in our environment [3]. Hence, although a conscious use of resources and effective waste reduction and management strategies are the primary way to a green future, bioplastics can represent a valid ally in the pathway towards a more sustainable society.

Despite their promising properties, the bioplastics market currently represents only a minor fraction of global plastics production. In 2021 the worldwide bioplastic production was 2.42 million tons out of the 367 million tons of plastics produced annually [4], i.e., still less than 1%. Nevertheless, the growth of the bioplastics market is expected to be significant in the coming years, as a response to the world’s urgent environmental needs. The main reasons for the limited application of bioplastics stem from the generally higher cost and lower thermo-mechanical performance compared to oil-based plastics, and therefore a considerable research effort is still needed to improve the properties of these materials while tailoring their biodegradation rate, to expand the applicability of bioplastics in many other fields. Only in this way, will the replacement of conventional plastics with bioplastics be truly sustainable [5]. 

One of the most widely used and promising biopolymers is poly(lactic acid) or polylactide (PLA). Its combination of high elastic modulus (2–4 GPa) and strength (30–50 MPa), high optical transparency, and compostability make it the ideal material for packaging applications, especially for food and other perishable substances [6,7,8,9]. However, PLA is generally used only for rigid thermoformed packaging [10,11,12,13], due to its poor strain at break (1–5%) and impact strength, very limited UV shielding capability, and inadequate gas-barrier properties [14]. PLA’s brittleness has been mitigated by adding low-molecular-weight plasticizers, such as citrate esters [15] and poly(alkylene glycol)s [16,17], or tough polymers, such as poly(ε-caprolactone) [18,19] or polybutylene adipate-co-terephthalate [20,21,22], but their impact on PLA’s stiffness and strength is, in most cases, deleterious [23,24]. Hence, although relevant steps have recently been taken through the development of blends and copolymers of PLA and other bioderived building blocks [25], finding a suitable, largely available, and cost-effective biobased additive for PLA that improves its ductility without impairing its stiffness and strength is still an open research challenge. 

An attractive class of biopolymers that can be blended with PLA is indeed that of the furanoate polyesters or poly(alkylene furanoate)s (PAFs). These polymers embody a viable biobased alternative to petrochemical-derived terephthalate polyesters and are synthesized from furan-2,5-dicarboxylic acid (FDCA). FDCA, ranked among the top 12 green building-block chemicals derived from the fermentation of carbohydrates [26], is characterized by a five-membered aromatic ring with one oxygen atom and can be polymerized with ethylene glycol into a fully bioderived poly(ethylene 2,5-furandicarboxylate) (PEF). This polymer has recently received increasing attention not only for the lower energy use and CO_2_ emissions in the initial phases of the life cycle compared to poly(ethylene terephthalate) (PET), but also for its very promising mechanical, thermal, optical, and gas-barrier properties, in some cases even higher than those of PET, which would also allow for a decrease of the carbon footprint of PEF-based materials in their use phase [27,28,29,30].

Notwithstanding the increasing number of research works on furanoate polyesters, the corpus of studies on furanoate-based polymer blends is still limited. Blends among furanoate polyesters such as PEF/PBF, PEF/PPF, and PBF/PPF were found to be fully miscible [31], while PAFs and their petrochemical counterparts (poly(alkylene furanoate)s, PATs) are poorly miscible if produced by solvent mixing and partially miscible if processed from the melt [32,33]. For blends of PLA and furanoate polyesters, the most investigated blend is PLA/PBF, reported as immiscible but with interesting mechanical properties if the weight fraction of PBF is kept under 5 wt % [34,35].

Our group has recently performed a thorough investigation on the physical-mechanical properties of PLA/PAF blends prepared via solution mixing, for the development of bioderived films [36,37,38,39,40] and fibers [41,42,43], involving not only PBF but also longer-alkyl-chain PAFs such as poly(pentamethylene furanoate) (PPeF), poly(hexamethylene furanoate) (PHF), poly(octamethylene furanoate) (POF), poly(decamethylene furanoate) (PDeF), and poly(dodecamethylene furanoate) (PDoF). These works have confirmed that a small fraction of such PAFs can considerably enhance the strain at break and the fracture toughness of neat PLA. However, the scarce adhesion between the PAF domains and the surrounding PLA matrix have led us to infer that even better properties could be obtained upon the addition of a proper compatibilizer. 

The literature abounds with studies on the compatibilization of PLA-based blends. Among the investigated compatibilizers, the most widely used are glycidyl ester materials. Thanks to their highly reactive epoxy groups, these compounds promote the formation of copolymers among the two blended polymers [44]. Therefore, they can effectively decrease the size of the dispersed phase, promote its uniform distribution in the continuous phase, and increase the interfacial adhesion, thereby enhancing the overall physical-mechanical properties [45]. Such glycidyl esters can also be used as chain extenders, as they also promote the formation of covalent bonds among PLA chains. This helps retain the molecular weight and counteract the degradation due to processing temperatures, thus improving the melt strength and extending the processing window of PLA-based materials [46]. 

Other problems that have emerged during the previous works of our group derived from the solution blending process. Although solution blending allows for work at low temperatures, thus preventing unwanted transesterification reactions typically observed with furanoate polyesters [27], it has several drawbacks, as it is poorly scalable and not very environmentally friendly, and some residual solvent can remain in the samples and affect the resulting thermomechanical properties. On the other hand, investigating the properties of PLA/PAF blends through melt mixing can produce industrially relevant results, applicable not only in industrial manufacturing plants but also in large-scale recycling facilities. 

Hence, this work aims to investigate, for the first time, the properties of biobased PLA/PEF blends produced via melt mixing as a function of their composition (i.e., the PEF fraction) and the amount of a commercial compatibilizer/chain extender. The work was divided into three main steps. After a preliminary comparison among four commercial compatibilizers (results not shown) (i), the most effective compatibilizer (Joncryl ADR4468, J) was selected and employed to prepare PLA/PEF blends with a variable J content (0.25 to 1 phr) and a fixed PEF weight fraction (i.e., 10 wt %), to identify the optimal J content (ii). This J content was then kept constant and the properties of the compatibilized blends were studied as a function of the PEF fraction (1 to 30 wt %) (iii). The characterization involved the study of the blends’ microstructure, thermal properties, resistance to thermal degradation, mechanical performance, optical transparency, and oxygen transmission rate. 

## 2. Results and Discussion

### 2.1. Choice of the Compatibilizer

The first part of the activity was devoted to choosing the most promising compatibilizer/chain extender. Given the scarce availability of PEF, the choice of the compatibilizer was made by mixing neat PLA with up to 3 phr of each compatibilizer and characterizing the resulting materials from the rheological and mechanical point of view (data not reported for brevity). It emerged that the addition of only 0.25 phr of compatibilizer Joncryl (J) positively modified the complex viscosity and the mechanical performance of PLA, considerably more than all the other investigated compatibilizers added in a weight fraction of up to 3 phr (see Materials and Methods Section 3 for details on the compositions of the characterized blends). It was concluded that, at the selected processing conditions, J is more reactive than the other considered compatibilizers, and therefore it was chosen as the most promising compatibilizer/chain extender to prepare PLA/PEF blends. This work presents only the results relative to the samples containing J.

### 2.2. Dynamic Rheological Properties

**Figure 1:**
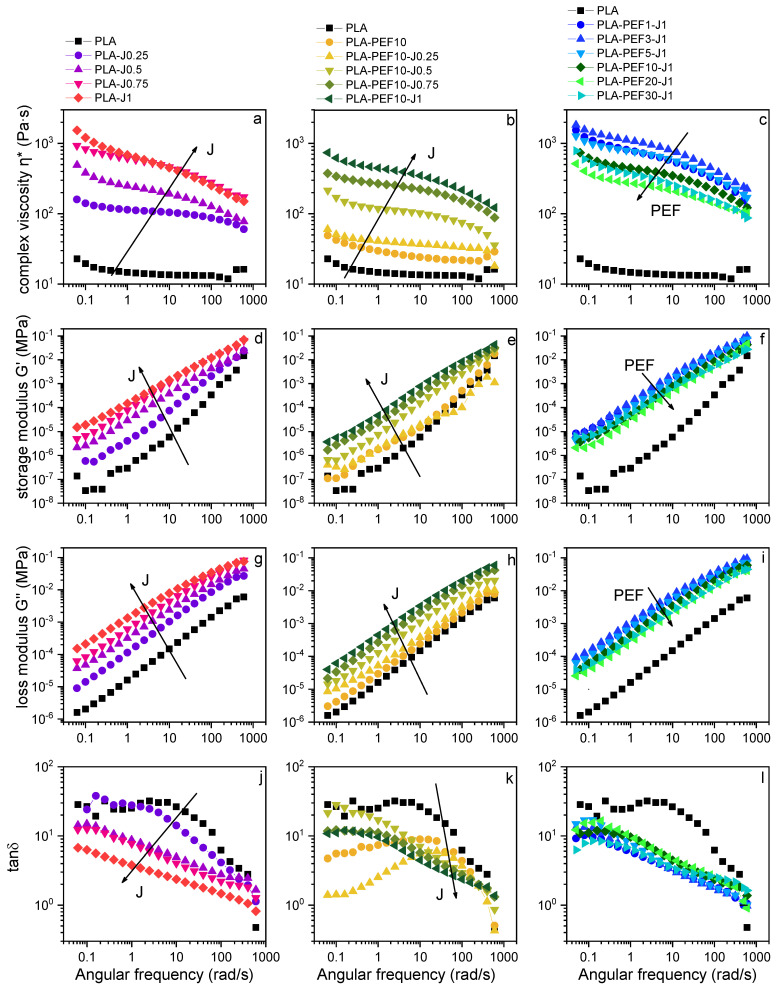
Results of the dynamic rheological tests at 230 °C on the prepared samples. Complex viscosity (**a**–**c**), storage modulus (**d**–**f**), loss modulus (**g**–**i**), and tanδ (**j**–**l**) as a function of the applied frequency for three series of samples.

Dynamic rheological tests were carried out to assess the individual and combined effect of PEF and J on the rheological properties of PLA at the processing temperature (230 °C). Figure 1a–l shows the results of the dynamic rheological tests performed on all the prepared blends. Here, the four main rheological parameters, i.e., complex viscosity, storage modulus, loss modulus, and tanδ are reported as a function of the J content in PLA (samples PLA-Jx, Figure 1a,d,g,j), as a function of the J content in the blend PLA-PEF10 (samples PLA-PEF10-Jx, Figure 1b,e,h,k), and as a function of the PEF content in the samples PLA-PEFx-J1 (Figure 1c,f,i,l). The parameters measured on neat PLA are reported everywhere, for reference. 

The complex viscosity of neat PLA is lower than what is reported in the literature for this or similar PLA grades [47,48]. This is the sum of two factors, i.e., (i) the degradation and decrease in molecular weight during processing, and (ii) the high testing temperature. The addition of J determines a considerable increase in the complex viscosity (Figure 1a) and the storage and loss modulus (Figure 1b–g), which increases by one order of magnitude with a J content of 0.25 phr and by two orders of magnitude with a J content of 1 phr. This implies that J is effective as a chain extender on neat PLA and helps counteract the decrease in molecular weight produced by the high-temperature processing needed to overcome the PEF’s melting temperature (218 °C, see Section 2.4). In fact, while the neat PLA evidences the general behavior of linear polymers with a rapid chain relaxation, in the samples PLA-Jx (x = 0.25–1) the Newtonian plateau progressively disappears, denoting an increased frequency sensitivity and marked shear thinning behavior, and this is an unambiguous sign of chain extension and enhanced entanglement [46]. Moreover, the decrease in the tanδ values (Figure 1j) with an increased J amount also denotes an increase in the melt elasticity. Due to the multifunctionality of J, each J molecule can react with more than two PLA chains, thereby producing a long-chain branched (LCB) structure [46]. However, the values of tanδ are practically never lower than 1, which indicates that the loss modulus is always greater than the storage modulus, in turn highlighting the persistence of a viscous, liquid-like behavior.

The addition of 10 wt % of PEF to neat PLA increases its complex viscosity and storage and loss modulus (Figure 1b,e,h), and this increase is more remarkable at lower frequencies. This effect is due to the influence of the interfacial interaction between the PEF droplets and the surrounding PLA and may indicate some compatibility [49]. This phenomenon is enhanced by adding increased amounts of J, which implies that J increases the interfacial interaction between the two polymer phases. On the other hand, the increase in PEF fraction given the same amount of J makes the compatibilization less effective, and therefore the complex viscosity and the storage and loss moduli (Figure 1c,f,i) decrease by increasing the PEF fraction; however, further investigation is needed to clarify this concept. 

### 2.3. Microstructural Properties

**Figure 2:**
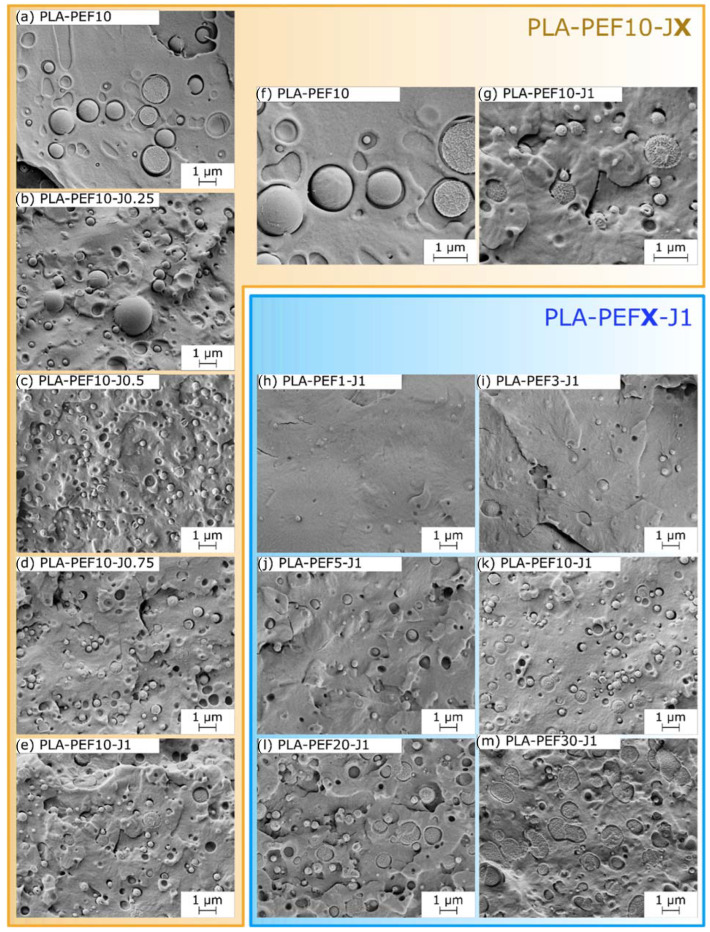
SEM micrographs of the cryofracture surface of the prepared samples. (**a**–**e**) PLA-PEF10-Jx (x = 0–1 phr); (**f**,**g**) comparison between PLA-PEF10 and PLA-PEF10-J1, at higher magnification; (**h**–**m**) PLA-PEFx-J1 (x = 1–30 wt %).

Figure 2a–m shows the SEM micrographs of the cryofracture surface of the prepared blends. In all samples, PEF is present as spheroidal domains, clearly distinguishable from the surrounding PLA matrix, but the size and shape of these domains vary considerably as a function of the relative amounts of PEF and J. In the uncompatibilized PLA-PEF10 blend (Figure 2a), PEF domains are micrometric and spherical and the interfacial adhesion with PLA is rather poor, as observable from the interfacial debonding. Most of the domains are smooth and intact, which implies that the fracture propagates mainly following the interfaces. 

**Figure 3:**
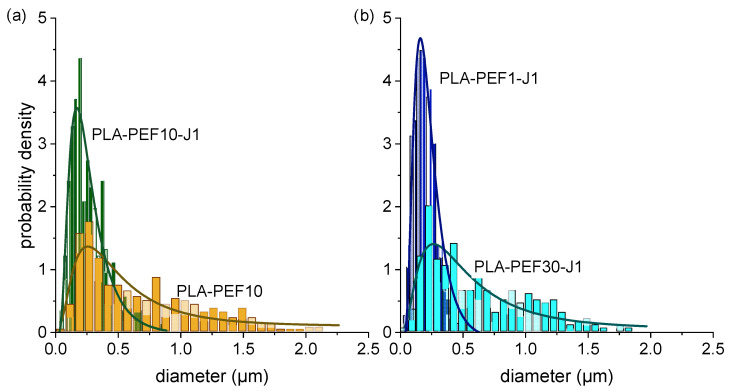
Diameter distribution of PEF domains for some selected compositions. Experimental values (histograms) and log-normal fitting (solid lines). (**a**) Effect of J: comparison between PLA-PEF10 and PLA-PEF10-J1 samples; (**b**) effect of PEF: comparison between PLA-PEF1-J1 and PLA-PEF30-J1 samples.

The addition of J (Figure 2b–e) reduces considerably the PEF domain size and increases the interfacial interaction with the PLA matrix, and these effects are more evident at higher J concentrations. From the PEF domain size distributions in Figure 3a and Table 1 (experimental and log-normally fitted values), the PEF domain size is seen decreasing from 0.67 ± 0.46 µm of PLA-PEF10 to 0.26 ± 0.14 µm of PLA-PEF10-J1, with a remarkable reduction not only of the average size but also of the size dispersion. This effect, together with the increased interfacial adhesion observable especially at higher magnifications (Figure 2f–g), is a clear sign of the positive compatibilizing function promoted by J [50]. 

On the other hand, the increase in PEF loading has the opposite effect (Figure 2h–m), since the domain size and the size dispersion considerably increase with the PEF concentration. At the highest investigated PEF concentration (PLA-PEF30-J1, Figure 2m), the PEF domains lose their spherical shape and start to coalesce, though keeping a good interfacial adhesion with the surrounding PLA. In this sample, the domain size is 0.62 ± 0.40 µm, comparable with that of the uncompatibilized PLA-PEF10, even though the PEF concentration here is three times higher, which demonstrates the effective compatibilization promoted by 1 phr of J even at higher PEF concentrations. 

**Figure 4:**
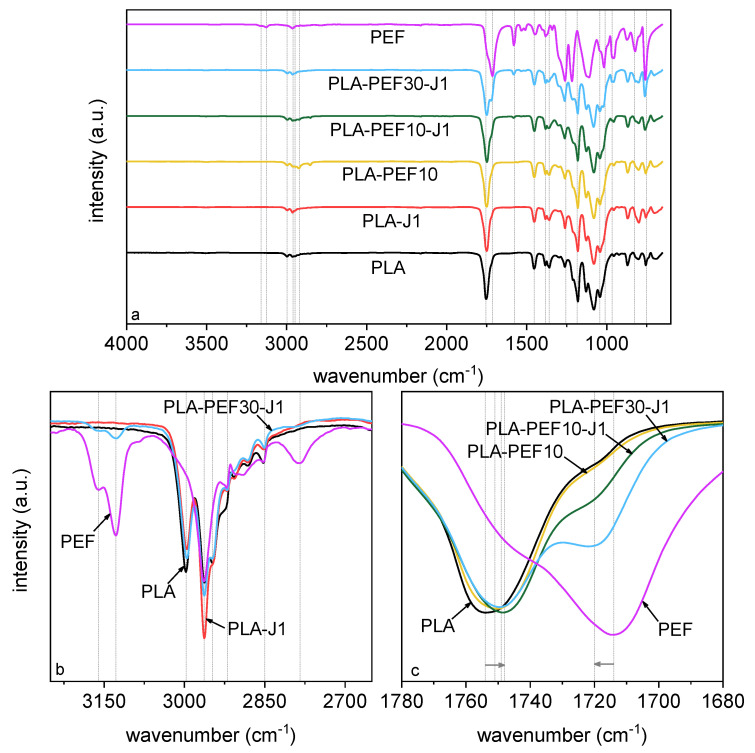
Results of the FT-IR analysis on some selected compositions. (**a**) Full spectrum; (**b**,**c**) highlights of some specific regions.

Figure 4a–c shows the FT-IR spectra of some selected samples, while Table 2 reports the main band assignments for the samples PLA, PEF, and PLA-PEF30-J1. All the most important signals can be appreciated already from the full IR spectra (Figure 4a) and are presented in detail in Figure 4b,c. The spectrum of neat PLA is coherent with the structure of this polymer, as it shows a weak signal of the in-plane and out-of-plane C–H stretching vibration in the interval 2950–3000 cm^−1^, of the C=O stretching vibration at 1754 cm^−1^, and of the C–O–C stretching vibration at 1179 cm^−1^ [51]. Neat PEF, on the other hand, shows the typical signals of furan-based aliphatic polyesters [52,53], i.e., the symmetric and asymmetric stretching of the furan ring at 3128 and 3163 cm^−1^, the symmetrical and asymmetrical C–H stretching vibration at 2963 and 2850 cm^−1^, the vibration of the C=C bond of furan ring at 1574 and 1530 cm^−1^, the carbonyl stretching vibration C=O, typical of ester groups, at 1714 cm^−1^ [54,55], and the furan ring breathing at 1016 cm^−1^ and ring bending at 969 cm^−1^, 827 cm^−1^, and 751 cm^−1^. The spectra of the blends show the same vibrations of the two neat polymers. If the bands at higher (>3000 cm^−1^) and lower (<1500 cm^−1^) wavenumbers are found in the same position as those of the neat polymers (Figure 4b, Table 2), the bands corresponding to the C=O stretching vibration evidence small red- or blue-shifts (Figure 4c), which may be due to some chemical interaction between the two phases. However, the contributions of both PLA and PEF are still well visible in the blends, which implies scarce homogenization [27].

### 2.4. Thermal Properties

**Figure 5:**
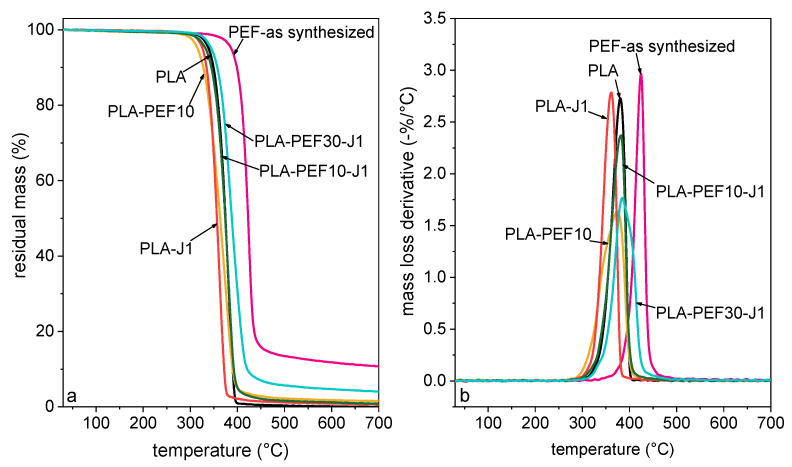
Representative TGA thermograms of some selected compositions. (**a**) Residual mass as a function of temperature; (**b**) mass loss derivative as a function of temperature.

Figure 5 shows the TGA thermograms of some selected compositions, while the most important TGA results are collected in Table 3. None of the samples show low-temperature mass loss associated with water absorption, which is a signal of the well-executed storage of the samples in dry conditions. Neat PLA degrades in a single step at approx. 380 °C, while the degradation of as-synthesized PEF occurs at a higher temperature, i.e., approx. 424 °C. The addition of J and PEF does not significantly vary the thermal degradation performance, although an increasing residual mass can be detected with an increase in the PEF concentration. 

**Figure 6:**
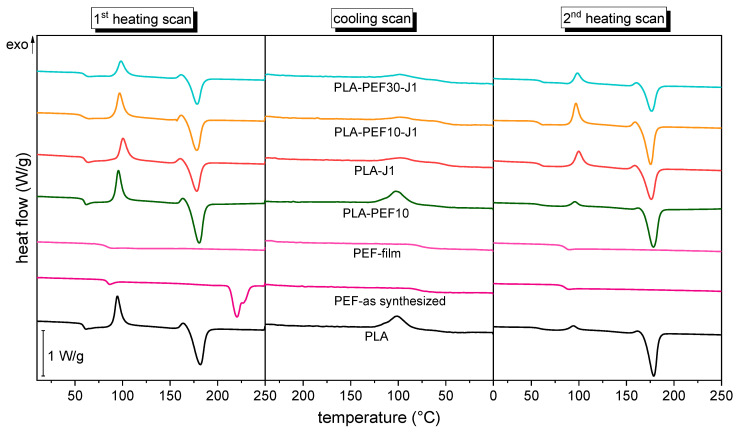
Representative DSC thermograms of some selected compositions. First heating scan, cooling scan, and second heating scan.

Figure 6 presents the DSC thermograms of some selected samples (first heating scan, cooling scan, and second heating scan), while the most important DSC results of all samples are reported in Table 4. The DSC thermograms of the first heating scan of neat PLA present the conventional profile of semicrystalline polymers, with an endothermic baseline deviation related to glass transition at approx. 56–58 °C, followed by an exothermic signal (approx. 100 °C) related to cold crystallization, and a final endothermic phenomenon (approx. 175 °C) associated with the melting of the crystalline phase. The cooling scan presents an exothermic signal at approx. 105 °C, related to the crystallization from the melt, while the second heating scan presents the same signals detected in the first, with a less remarkable cold-crystallization event and a more prominent melting peak, both associated with a higher crystallinity degree. The addition of increasing amounts of J to PLA slightly increases the *T_g_,* which could imply that J successfully counteracts the degradation by chain scission due to high-temperature processing [47]. On the other hand, the addition of J leaves the values of all the melting and crystallization temperatures generally unaltered, but, at high J fractions (≥0.75 phr), the crystallinity degree decreases, passing from 16 % of neat PLA to 8 % of the sample PLA-J1. This is evident only at higher J concentrations, due to the already low crystallization kinetics of such PLA types [56]. This should be noted since a lower crystallinity degree is associated not only with a lower tensile modulus and strength and higher strain at break, but also with worse gas-barrier properties [57].

For neat PEF, it is interesting to compare the as-synthesized material and the film sample produced by compression molding, both shown in Figure 6 and Table 4. In the first heating scan, the as-synthesized PEF shows a glass transition endothermic signal at 83 °C, a melting peak at 218 °C, and a crystallinity degree of 36%, in good agreement with what has been reported previously on similar PEF grades [58]. On the other hand, the cooling and the second heating scans present only the signals related to the glass transition, which suggests the absence of the crystalline phase after a cooling/heating rate of 10 °C/min. The portrait of a fully amorphous PEF with *T_g_* at 80 °C is also what emerges from the analysis of the PEF film sample, which implies that the cooling rate imposed in the hot-plate press was too high to let the polymer chains organize in the long-range order structures typical of the crystalline phase. 

The addition of 10 wt % PEF to PLA (sample PLA-PEF10) does not significantly shift the glass transition temperature, a symptom of scarce miscibility between the two phases. On the other hand, the crystallinity degree of the PLA phase increases to 28% (+72% than neat PLA), as already observed in previous works on other polylactide/poly(alkylene furanoate) blends with longer alkyl chains [39,42,59]. For the blends containing both PEF and J (samples PLA-PEF10-Jx and PLA-PEFx-J1), PEF and J have opposite effects on PLA’s crystallinity, as an increasing amount of J hinders the formation of highly crystalline phases while an increasing PEF fraction enhances the crystallization effect. 

**Figure 7:**
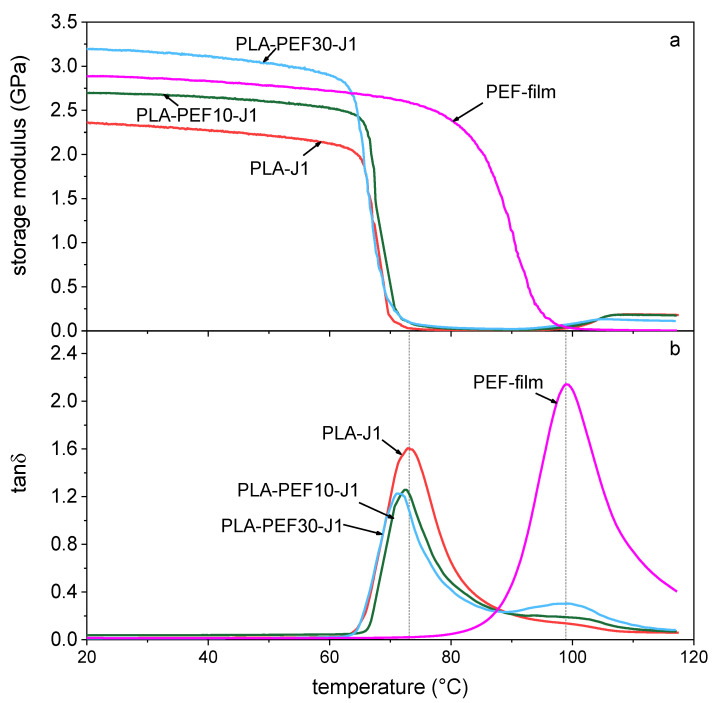
Representative DMTA thermograms of some selected compositions. (**a**) Storage modulus as a function of temperature; (**b**) tanδ (smoothed) as a function of temperature. Dashed vertical lines evidence the tanδ peak temperatures of PLA (73.2 °C) and PEF (98.8 °C).

Figure 7 shows the results of the DMTA tests, i.e., the storage modulus (Figure 7a) and the tanδ (Figure 7b) of some selected samples as a function of temperature. The glass transition temperature of PLA-J1, highlighted as the peak of the tanδ signal, is found at 73.1 °C, while that of neat PEF is at 98.8 °C. The corresponding signals of the blends are found in a very similar position as those of the two neat polymers, and this is evident not only for the $T_{g}$ of the PLA phase, already observed via DSC, but also for the PEF phase. This confirms the immiscibility of the two polymers. Moreover, the slight increase of the storage modulus of some compositions indicates the crystallization of the PLA phase of the investigated blends.

### 2.5. Mechanical Properties

**Figure 8:**
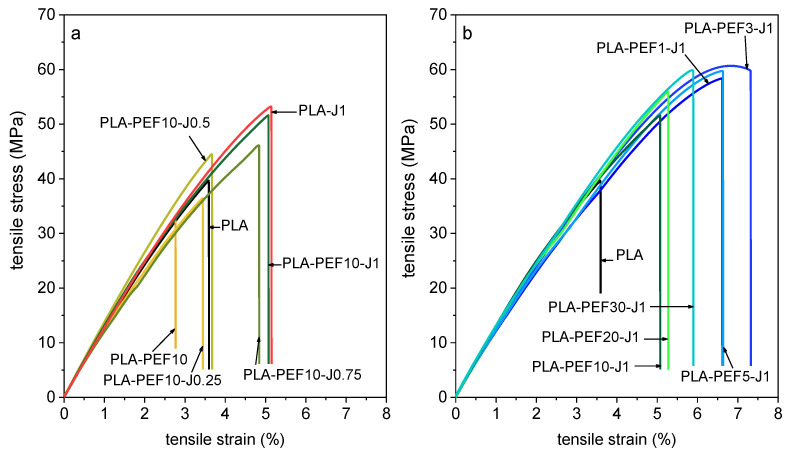
Representative stress–strain curves of some selected compositions. (**a**) PLA, PLA-PEF10-Jx (x = 0.25–1) and PLA-J1; (**b**) PLA and PLA-PEFx-J1 (x = 1–30).

**Figure 9:**
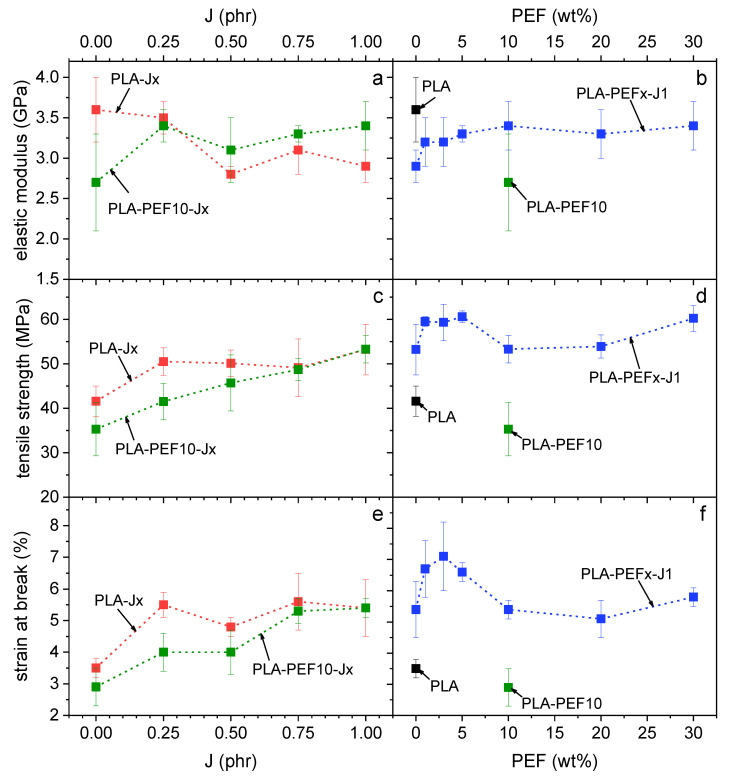
Main results of the tensile tests on the prepared samples as a function of the J content (**a**,**c**,**e**) and of the PEF weight fraction (**b**,**d**,**f**). (**a**,**b**) Elastic modulus; (**c**,**d**) tensile strength; (**e**,**f**) strain at break.

Figure 8a,b shows representative stress–strain curves obtained in quasi-static tensile tests for some selected compositions, while the most important results are presented in Figure 9a–f. Neat PLA shows an elastic modulus of approx. 3.6 GPa, a tensile strength of 41 MPa, and a strain at break of 3.5%, in line with semicrystalline PLAs of similar grade and in good agreement with the technical datasheet. The addition of J to neat PLA (Figure 9a,c,e, red dots) progressively decreases the elastic modulus and increases the properties at break, in agreement with the decrease in the degree of crystallinity measured via DSC. On the other hand, the addition of 10 wt % of PEF to neat PLA produces an uncompatibilized blend with lower mechanical properties than neat PLA, although the partial overlapping of the dispersion bands suggests a modest significance of this difference (Figure 9a,c,e, *J* = 0 phr). In any case, the elastic modulus decreases on average from 3.6 GPa to 2.6 GPa (−28%), the tensile strength from 41 MPa to 35 MPa (−15%), and the strain at break from 3.5% to 2.8% (−20%). The addition of J to this blend improves all the investigated properties (Figure 9a,c,e), as expected from the finer microstructure and the increased PEF/PLA interfacial interaction detected in the compatibilized blends. The best performer, PLA-PEF10-J1, recovers the high elastic modulus of neat PLA and also exhibits an improved mechanical strength (53 MPa, +29%) and strain at break (5.5%, +57%). 

For the blends with a fixed J content (1 phr) and a varying PEF fraction (1–30 wt %) (Figure 9b,d,f), the elastic modulus generally increases with the PEF content, probably due to a combination of a high elastic modulus of PEF and the higher crystallinity degree of the PLA phase. On the other hand, the strain at break (and to some extent also the tensile strength) show a maximum at PEF fractions of 3–5 wt %, for which the stress–strain curve manifests an incipient yield point (Figure 8b), probably due to the plasticization effect of the amorphous PEF domains. A similar effect has also been observed by Long et al. [35] for PLA/PBF blends, even though in that case the increase in the strain at break was more remarkable, probably due to the higher flexibility of PBF compared to PEF. In any case, all the PLA/PEF compatibilized blends prepared in this work show higher tensile strength and strain at break than neat PLA, while retaining their high elastic moduli.

### 2.6. Functional Properties

**Figure 10:**
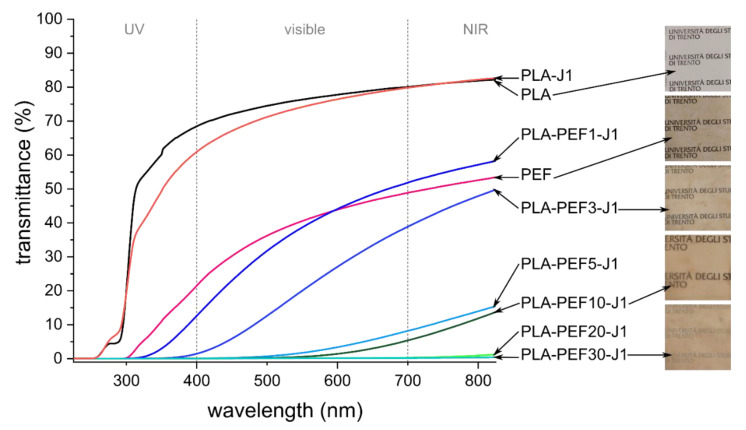
Transmittance spectra of the prepared films with pictures of some selected specimens (square edge = approx. 4 cm).

Transmittance tests were carried out as the optical transparency in the visible range can be a very interesting property, especially for packaging applications. Figure 10 shows the transmittance spectra of some selected film samples, together with some pictures of the films. Neat PLA and PLA-J1 feature high and comparable transmittance values thanks to the generally low degree of crystallinity, being approx. 65–80% in the visible range. These values are slightly lower than those reported in our previous works on PLA-based films [37,39] or in other works from the literature on similar materials [60], which stems from the higher film thickness in this work (i.e., 120 µm) compared to the previous works (i.e., 50 µm). 

Neat PEF film shows lower transmittance than neat PLA, although certain optical transparency is still retained at these thicknesses, as shown in the photograph reported in Figure 10. It is interesting to note that the transmittance in the UV region for the neat PEF film is remarkably lower than that of neat PLA, due to the UV absorption capacity of furan rings conjugated with carbonyl groups [61]. A strong UV absorption is very positive for packaging applications as the UV light, already at 380 nm, has enough energy to induce autoxidation of fats, degradation of vitamins, and discoloration of fresh meat, thereby lowering the food quality [60,62]. Therefore, the optimal food packaging material couples strong UV barrier properties with transparency in the visible range. 

It is very interesting to note that this combination of properties can be obtained by adding only 1 or 3 wt % of PEF to PLA, as already observed in our previous works with other poly(alkylene furanoate)s [37,39]. In fact, whereas the optical transparency decreases considerably with a PEF fraction higher than 5 wt %, the samples PLA-PEF1-J1 and PLA-PEF3-J1 retain good transparency in the visible range and exhibit remarkable UV-shielding properties, which makes these compositions very promising for packaging of food and other perishable items. 

**Figure 11:**
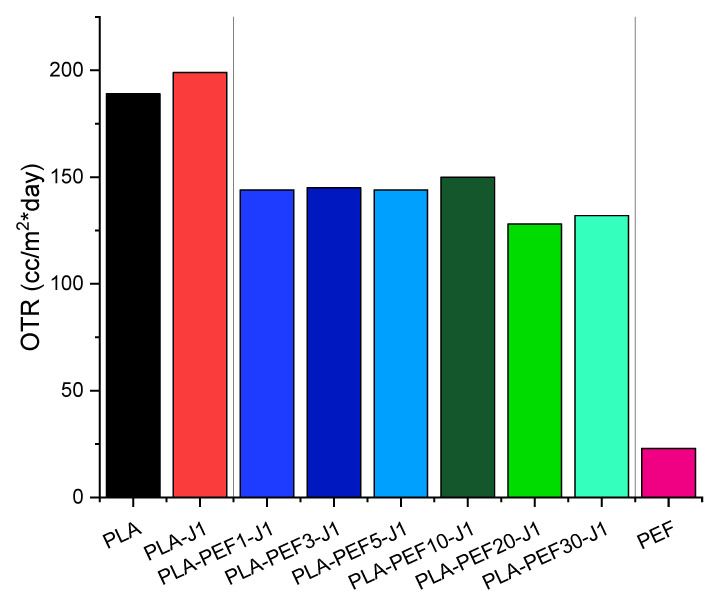
Results of the oxygen permeability test: values of the oxygen transmission rate (OTR) on some selected samples.

Finally, Figure 11 shows the OTR values for neat PLA, neat PEF, and PLA-PEF films with a fixed J content (1 phr) and a varying PEF fraction (1–30 wt %). As known, PLA shows a relatively high value of OTR (i.e., 189 cc/m^2^·days) thus confirming its scarce oxygen-barrier properties [14], whereas PEF exhibits noticeably higher barrier properties (i.e., 23 cc/m^2^·day) [63]. The addition of J leads to an increment of OTR in comparison to the neat PLA film. This result can be ascribed to the decrease of the crystallinity degree, which passes from 16% of neat PLA to 8% of the sample PLA-J1, as reported in Table 4. 

For PLA-PEF blends, the OTR values are quite encouraging because it is clear that all the investigated films present enhanced oxygen barrier properties when compared with neat PLA and PLA-J1 films. In particular, the addition of only 1 wt % PEF to PLA allows for the obtaining of OTR reductions of about 24% and 28% in comparison with PLA and PLA-J1 films, respectively. However, the oxygen barrier properties do not vary significantly by increasing the PEF content, although the PLA-based films with larger PEF content (i.e., 20% and 30%) show the lowest OTR values among the investigated blends, due both to the intrinsic barrier properties of PEF and the higher crystallinity degree shown by these samples. To reach very low OTR values, comparable with those of neat PEF, one should further increase the PEF content so as to reach a co-continuous microstructure. In any case, a small amount of PEF is still beneficial to the improvement of the gas-barrier properties of PLA. 

## 3. Materials and Methods

### 3.1. Materials

Poly(lactic acid) Ingeo^®^ 2500 HP was supplied by NatureWorks LLC (Minnetonka, MN, USA) in the form of granules (density = 1.24 g/cm^3^, melt flow rate at 210 °C and 2.16 kg = 8 g/10 min ) and used as received. High-molecular-weight poly(ethylene 2,5-furandicarboxylate) (PEF) was synthesized via a 2-stage melt polycondensation procedure. The resulting PEF had an intrinsic viscosity of 0.42 dL/g and after solid state polycondensation at 210 °C for 5 h was increased to 0.69 dL/g [64], which implies a molecular weight close to 30000 g/mol. The availability of PEF for this trial was approx. 63 g. The employed compatibilizer was Joncryl^®^ ADR 4468 (density = 1.08 g/cm^3^, glass transition temperature = 59 °C). Joncryl was purchased by BASF GmbH (Ludwigshafen am Rhein, Germany) in the form of flakes and used as received. The other three commercial compatibilizers were the DuPontTM Entira™ Strong 1002 polymer modifier (DuPont Packaging & Industrial Polymers, Wilmington, DE, USA), and the poly(ethylene-co-methyl acrylate-co-glycidyl methacrylate) (EMA-GMA) and poly(ethylene-co-glycidyl methacrylate) (E-GMA), both provided by Sigma-Aldrich (Saint Louis, MO, USA). 

### 3.2. Sample Preparation

Samples of PLA/PEF uncompatibilized and compatibilized blends were produced via melt compounding and hot-pressing in the form of sheets (2 mm thick) and films (120 µm thick). Granules of PLA, PEF, and J were dried overnight at 80 °C in vacuum conditions and then melt-compounded in batches of 45 g in a Thermo Haake Rheomix 600 internal mixer equipped with counter-rotating rotors, operating at 60 rpm at a temperature of 230 °C for a total of 7 min (J was added at minute 3, after complete melting of PLA and PEF). Such a high compounding temperature was necessary in order to overcome the melting temperature of PEF. A preliminary thermogravimetric analysis (TGA) on the PLA granules confirmed that the processing temperature, although considerably higher than the common processing temperature of this PLA grade was below its degradation temperature. Nevertheless, the materials were melt-compounded for the minimum amount of time necessary to allow the reactive compatibilization promoted by Joncryl, which was monitored by the raising of the compounding torque until a plateau, at which the reactive compatibilization was considered complete.

The compounded blends were then compression molded in a Carver hot-plate press at 230 °C for 5 min, under an applied load of 10 tons, to obtain square sheets of 120 × 120 × 2 mm^3^. To obtain thin films, some pieces of these sheets were cut and further compressed at 230 °C in a mold of 80 × 80 × 0.12 mm^3^, under a load of 0.25 tons applied for 2 min. Thin films of PEF were also produced by starting from the synthesized granules, while it was not possible to produce a 2-mm-thick sheet of neat PEF due to the scarcity of material available.

The composition of the prepared blends, in terms of the relative amount of PEF and J, was selected to investigate first the effect of various amounts of compatibilizer and then the effect of an increasing amount of PEF. Hence, samples were prepared with a fixed amount of PEF (i.e., 10 wt %) and a variable amount of J, ranging from 0.25 to 1 phr. Then, other samples were prepared by fixing the amount of J (i.e., 1 phr) and varying the PEF fraction (from 1 to 30 wt %). Neat PLA/J samples without PEF were also prepared to isolate the effect of J on PLA. The list of prepared samples is shown in Table 5.

### 3.3. Characterization

The rheological properties of the prepared blends were investigated with a Discovery HR-2 hybrid rheometer (TA instrument, New Castle, DE, USA), in a parallel-plate configuration with a gap distance of 2 mm. The tests were performed on discoidal specimens die-cut from the prepared sheets (thickness 2 mm, diameter 25 mm), in frequency sweep mode from 0.1 to 100 rad/s at 230 °C. The microstructural properties of the prepared samples were evaluated by analyzing the cryofracture surface with a field-emission scanning electron microscope (FE-SEM) Zeiss Supra 40 (Carl Zeiss AG, Oberkochen, Germany) after Pt-Pd sputtering. Fourier-transform infrared (FT-IR) spectroscopy was performed in attenuated total reflectance (ATR) configuration on the surface of the prepared sheets via a Perkin-Elmer Spectrum One instrument (Perkin Elmer GmbH, Waltham, MA, USA), equipped with a ZnSe crystal and operating in a wavenumber range 650–4000 cm^−1^. 100 scans were superimposed for each spectrum. The resolution of the instrument is 4 cm^−1^.

Thermogravimetric analysis (TGA) was performed with a Mettler TG50 thermobalance (Mettler Toledo, Columbus, OH, USA). Specimens of approx. 20 mg cut from the prepared sheets were heated at 10 °C/min up to 700 °C, under a nitrogen flow of 20 mL/min. One specimen was tested per composition. The tests allowed the measurement of the temperatures corresponding to a mass loss of 1 wt %, 3 wt %, and 5 wt % ($T_{1\%,\;}\;T_{3\%,\;}\;T_{5\%}$), the onset degradation temperature ($T_{onset}$), defined with the tangent method, the degradation temperature ($T_{d}$), corresponding to the peak of the derivative thermogravimetry (DTG) curve, and the final mass after the test ($m_{r}$). Differential scanning calorimetry (DSC) was performed with a Mettler DSC30 calorimeter (Mettler Toledo, Columbus, OH, USA) on specimens cut from the prepared sheets. Specimens of approx. 15 mg were subjected to a heating-cooling-heating cycle at 10 °C/min between 0 °C and 250 °C, under a nitrogen flow of 100 mL/min. One specimen was measured per sample. The test allowed for the measuring of the glass transition temperature ($T_{g}$) and the melting, crystallization, and cold crystallization temperatures and enthalpies ($T_{m,\;}\;T_{c,\;}\;T_{cc,\;}\;\Delta H_{m,\;}\;\Delta H_{c,\;}\;\Delta H_{cc}$) of the PLA and PEF phases. Moreover, the degree of crystallinity of PLA and PEF ($\chi_{PLA},\;\chi_{PEF}$) was calculated via Equation (1):

(1) $$\chi =\frac{\;\Delta H_{m}-\Delta H_{cc}}{\;\Delta H_{0}\cdot \omega }\cdot 100$$

where $\Delta H_{0}$ is the theoretical melting enthalpy, equal to 93.7 J/g for PLA [65] and 140 J/g for PEF [58], and *ω* is the weight fraction of PLA or PEF. Dynamic mechanical thermal analysis (DMTA) was carried out on some of the prepared films (i.e., PLA-J1, PLA-PEF10-J1, PLA-PEF30-J1, and PEF) to measure the glass transition temperature of the PEF phase in the blends. The tests were performed with a TA Q800DMA instrument (TA instrument, New Castle, DE, USA) in tensile mode on specimens of nominal dimensions 30 × 8 × 0.12 mm^3^ cut out of the prepared films. Storage modulus (E′), loss modulus (E″), and loss factor (tanδ) were measured between 20 and 120 °C, at a heating rate of 3 °C/min, at a strain amplitude of 0.05% and a frequency of 1 Hz.

The mechanical properties of the prepared blends were investigated via quasi-static tensile tests performed on dumbbell 1BA specimens (UNI EN ISO 527-2), laser-cut from the prepared sheets, via an Instron 5969 universal testing dynamometer (Norwood, MA, USA), equipped with a 1-kN load cell. To determine the elastic modulus (E), five specimens were tested at 0.25 mm/min while the strain was measured with a resistance extensometer Instron 2620–601, having a gauge length of 12.5 mm. The elastic modulus was evaluated as the slope of the stress–strain curve between the strain levels of 0.05 % and 0.25%. Five additional specimens were tested at 1 mm/min until rupture, and these properties allowed the measurement of the ultimate tensile strength (UTS), evaluated as the maximum stress, and the strain at break ($\varepsilon_{b}$).

Optical transmittance was measured to study variations in the transparency of the prepared films as a consequence of increasing PEF and J amounts. Tests were carried out with a JascoV-570 dual-beam spectrophotometer (Jasco, Easton, MD, USA). Values of transmittance were acquired in the UV-visible-near infrared (UV-Vis-NIR) range of 200–2500 nm with an acquisition speed of 400 nm/min and an excitation bandwidth of 2 nm. Finally, the oxygen transmission rate (OTR) of some prepared films (i.e., PLA, PLA-J1, PLA-PEF1-J1, PLA-PEF3-J1, PLA-PEF5-J1, PLA-PEF10-J1, PLA-PEF20-J1, PLA-PEF30-J1, and PEF) was evaluated through an Oxygen Permeation Analyzer model M8001 (Systech Illinois, Thame, UK), following the ASTM D3985 standard. Due to the limited availability of PEF, only one sample was tested for each composition. OTR values were acquired at 23 °C with 0% RH by using high purity oxygen gas as the testing gas (i.e., purity > 99.9%) and high purity nitrogen gas as the carrier gas (i.e., purity > 99.999%). The films were tested by using a specific mask to reduce the test area to 5 cm^2^.

## 4. Conclusions

This work investigated the thermal, mechanical, and functional properties of melt-mixed, fully bioderived blends of PLA and PEF as a function of the PEF weight fraction (1–30 wt %) and the amount of the commercial compatibilizer/chain extender Joncryl ADR 4468 (J, 0.25–1 phr). The first step was devoted to understanding the effects of an increasing amount of J on neat PLA and the blend PLA-PEF10, to select the most promising amount of J, while the second step was aimed at characterizing the properties of compatibilized PLA/PEF blends containing 1 phr of J and a variable PEF weight fraction. 

J was proven effective both as a compatibilizer and as a chain extender for this blend. J successfully mitigated the immiscibility of the two polymer phases by decreasing and homogenizing the PEF domain size; for the blend containing 10 wt % of PEF, the PEF domain size dropped from 0.67 ± 0.46 µm of the uncompatibilized blend to 0.26 ± 0.14 with 1 phr of J. Moreover, the increase in the complex viscosity of PLA and PLA/PEF blends with the J content evidenced the effectiveness of J as a chain extender, which helped counteract the decrease in molecular weight produced by the high-temperature processing needed to overcome the high PEF melting temperature (i.e., 218 °C).

DSC tests highlighted how PEF and J had opposite effects on PLA’s crystallinity, as an increasing amount of J hindered the formation of highly crystalline phases, while an increasing PEF fraction enhanced the crystallization. In the sample PLA-PEF10, the crystallinity degree of PLA increased to 28% (+72% than neat PLA), while in the sample PLA-J1 it dropped to 8% (−50% than neat PLA). This is important to point out, as a higher crystallinity degree generally translates into higher gas-barrier properties but lower optical transparency, both very important properties for packaging applications. 

The positive contribution of J emerged in the mechanical characterization. The uncompatibilized PLA-PEF10 blend showed poorer mechanical performance than neat PLA, with a lower elastic modulus, tensile strength, and strain at break. On the other hand, all the compatibilized blends showed higher tensile strength and strain at break than neat PLA, while retaining their high elastic moduli. The elastic modulus increased with the PEF fraction, up to 3.4 GPa for PLA-PEF30-J1, while the tensile strength and strain at break showed a maximum at 3–5 wt % of PEF. The sample with the most balanced property set is PLA-PEF3-J1, showing an elastic modulus of 3.2 GPa (−11% than PLA, +10% than PLA-J1), a tensile strength of 59.6 MPa (+42.5% than PLA), and a strain at break of 7.1% (+103% than PLA). 

The effect of PEF on the UV- and oxygen-barrier properties of PLA was also remarkable. Adding only 1 wt % of PEF made the blend an excellent barrier for UV rays, since the transmittance at 320 nm dropped from 52.8% of neat PLA to 0.4% of PLA-PEF1-J1, while keeping good transparency in the visible region. PEF was also responsible for a sensible decrease in the oxygen transmission rate, which passed from 189 cc/m^2^·day for neat PLA to 144 cc/m^2^·day with only 1 wt % of PEF. 

This work emphasized the synergistic effects of PEF and J in enhancing the thermal, mechanical, and functional properties of PLA, which resulted in bioderived blends that are very promising for packaging applications.

## Figures and Tables

**Table 1:** Mean and standard deviation values for the PEF domain size in the prepared samples. Experimental values and results of the log-normal fitting with the associated R^2^ value.

Sample	Domain Size (Experimental) (µm)	Domain Size (Log-Normal Distribution) (µm)	R^2^
PLA-PEF10	0.67 ± 0.46	0.57 ± 0.48	0.83
PLA-PEF10-J0.25	0.40 ± 0.29	0.41 ± 0.29	0.94
PLA-PEF10-J0.5	0.28 ± 0.12	0.30 ± 0.14	0.95
PLA-PEF10-J0.75	0.32 ± 0.14	0.37 ± 0.21	0.92
PLA-PEF10-J1	0.26 ± 0.14	0.28 ± 0.17	0.91
PLA-PEF1-J1	0.21 ± 0.09	0.23 ± 0.13	0.96
PLA-PEF3-J1	0.25 ± 0.12	0.25 ± 0.09	0.94
PLA-PEF5-J1	0.28 ± 0.14	0.28 ± 0.14	0.96
PLA-PEF20-J1	0.53 ± 0.32	0.65 ± 0.55	0.86
PLA-PEF30-J1	0.62 ± 0.40	0.63 ± 0.56	0.79

**Table 2:** Results of FT-IR analysis. Band position (cm^−1^) and assignment of the main signals from the sample PLA, PEF, and PLA-PEF10.

Assignment	PLA	PEF	PLA-PEF30-J1
ν_o.p_. CH - Fu	-	3163	3163
ν_i.p._ CH - Fu	-	3128	3128
ν_o.p_. CH_3_	2997	-	2996
ν_i.p._ CH_3_	2948	-	2948
ν_o.p._ CH	2963	2963	2963
ν C = O	1754	1714	1748; 1720
ν C = C - Fu	-	1580	1583
ν C - O	1179	1217	1179; 1217
Fu ring breathing	-	1016	1021
Fu ring bending	-	969; 827; 751	969; 823; 762

Fu—furan ring; ν—stretching vibration; o.p.—out-of-phase; i.p.—in-phase.

**Table 3:** Main results of the TGA tests on the prepared samples.

Sample	$T_{1\%\;}$ (°C)	$T_{3\%\;}$ (°C)	$T_{5\%\;}$ (°C)	$T_{onset\;}$ (°C)	$T_{d\;}$ (°C)	$m_{r\;}$ (%)
PLA	305.1	330.6	339.1	355.5	380.3	0.0
PEF-as received	324.7	375.6	387.0	402.4	424.5	10.7
PLA-J0.25	310.9	331.0	338.4	352.8	379.2	0.0
PLA-J0.5	289.1	325.1	334.3	349.9	375.0	0.0
PLA-J0.75	319.7	336.5	343.0	355.0	379.5	0.0
PLA-J1	296.9	318.3	325.9	336.3	360.8	0.5
PLA-PEF10	272.4	306.7	315.6	328.6	374.2	1.6
PLA-PEF10-J0.25	255.9	320.8	331.2	348.4	376.0	0.5
PLA-PEF10-J0.5	300.8	326.3	334.6	354.8	380.2	1.0
PLA-PEF10-J0.75	290.1	326.4	335.3	354.1	380.9	0.9
PLA-PEF10-J1	285.2	321.9	332.6	357.3	382.0	0.8
PLA-PEF1-J1	297.1	329.7	338.3	354.5	379.3	0.0
PLA-PEF3-J1	313.2	334.7	342.1	356.4	380.2	0.3
PLA-PEF5-J1	313.3	333.1	340.8	356.9	380.5	0.4
PLA-PEF20-J1	322.0	339.1	347.1	360.5	387.7	2.7
PLA-PEF30-J1	309.9	335.1	344.7	363.1	386.3	4.1

$T_{1\%},\;T_{3\%\;},\;T_{5\%\;}$ = temperatures corresponding to a mass loss of 1%, 3%, and 5%;$\;T_{onset}=$ onset degradation temperature; $\;T_{d}=$ peak degradation temperature; $\;m_{r}=$ residual mass after the test.

**Table 4:** Main results of the DSC tests on the prepared samples. All properties refer to the PLA phase, except for the samples composed of neat PEF (*).

Scan	First Heating Scan						Cooling Scan		Second Heating Scan					
Property/ Sample	$T_{g\;}$ (°C)	$T_{cc\;}$ (°C)	$\Delta H_{cc\;}$ (J/g)	$T_{m}$ (°C)	$\Delta H_{m\;}$ (J/g)	χ (%)	$T_{c\;}$ (°C)	$\Delta H_{c\;}$ (J/g)	$T_{g\;}$ (°C)	$T_{cc\;}$ (°C)	$\Delta H_{cc\;}$ (J/g)	$T_{m}$ (°C)	$\Delta H_{m\;}$ (J/g)	χ (%)
PLA	58.1	97.7	40.4	178.6	55.9	16.5	104.0	42.2	56.8	96	3.1	175.4	55.7	56.1
PEF-as synthesized	83.6 *	-	-	218.4 *	49.9 *	35.6 *	-	-	84.3 *	-	-	-	-	0.0 *
PEF-film	82.3 *	-	-	-	-	0.0 *	-	-	84.8 *	-	-	-	-	0.0 *
PLA-J0.25	59.8	96.9	35.9	179.1	57.7	23.3	101.2	32.5	59.0	95.9	9.3	176.1	55.7	49.6
PLA-J0.5	59.6	97.5	31.3	177.7	47.9	17.8	101.9	28.2	58.0	96.7	8.32	174.6	48.6	43.2
PLA-J0.75	59.5	99.6	36.4	178.3	49.5	14.1	98.7	9.0	60.2	99.6	26.1	175.4	50	25.7
PLA-J1	59.7	100.7	31.5	176.3	38.9	8.0	98.9	10.1	58.9	99.1	24.1	174.2	43.2	20.6
PLA-PEF10	59.2	96.0	31.7	178.6	55.6	28.3	103.6	36.2	58.0	95.5	5.1	176.5	52.4	56.1
PLA-PEF10-J0.25	55.3	96.3	37.7	178.8	49.7	14.3	100.8	19.2	57.5	96.4	17.7	174.8	50.6	39.1
PLA-PEF10-J0.5	58.7	96.3	27.1	176.8	43.2	19.2	100.7	27.1	58.3	95.5	7.2	176.7	44.1	44.0
PLA-PEF10-J0.75	59.5	98.3	35.1	177.2	39.8	5.6	99.3	8.4	58.6	98.5	24.8	173.9	45.9	25.2
PLA-PEF10-J1	56.8	97.1	33.1	176.6	42.1	10.8	99.8	7.21	57.7	97.1	23.9	173.7	46.1	26.6
PLA-PEF1-J1	60.8	99.6	28.5	176.9	45.0	18.0	-	-	56.0	100.5	28.8	175.1	44.5	17.1
PLA-PEF3-J1	64.4	98.6	24.9	175.7	44.2	21.4	97.8	4.4	59.1	100.3	26.5	174.0	44.1	19.6
PLA-PEF5-J1	61.6	98.7	30.3	176.5	43.7	15.2	98.4	5.6	59.8	100.1	27.1	174.4	43.7	18.8
PLA-PEF20-J1	59.6	98.7	24.1	177.1	41.6	23.6	96.9	3.7	59.4	99.3	24.1	174.7	40.6	22.2
PLA-PEF30-J1	59.3	98.3	19.3	176.5	35.2	24.5	99.4	4.9	60.0	98.1	13.8	174.6	33.8	30.8

$T_{g\;}$= glass transition temperature; $T_{cc\;},\;\Delta H_{cc\;}$ = cold crystallization temperature and enthalpy; $T_{m\;},\;\Delta H_{m\;}$ = melting temperature and enthalpy; $T_{c\;},\;\Delta H_{c\;}$ = crystallization temperature and enthalpy; χ = crystallinity degree; - = not detectable. * = property related to the PEF phase.

**Table 5:** List of the prepared samples with nominal weight compositions.

Label	PLA (wt %)	PEF (wt %)	J (phr)
PLA	100	0	0
PLA-J0.25	100	0	0.25
PLA-J0.5	100	0	0.5
PLA-J0.75	100	0	0.75
PLA-J1	100	0	1
PLA-PEF10	90	10	0
PLA-PEF10-J0.25	90	10	0.25
PLA-PEF10-J0.5	90	10	0.5
PLA-PEF10-J0.75	90	10	0.75
PLA-PEF10-J1	90	10	1
PLA-PEF1-J1	99	1	1
PLA-PEF3-J1	97	3	1
PLA-PEF5-J1	95	5	1
PLA-PEF20-J1	80	20	1
PLA-PEF30-J1	70	30	1
PEF	0	100	0

## Data Availability

Data are available on request by the corresponding author.

## Sample Availability

Samples of the produced compounds are available from the authors.
